# Giant cell arteritis during COVID-19 pandemic

**DOI:** 10.5935/0004-2749.202100114

**Published:** 2021

**Authors:** Luciano Simão, André Messias

**Affiliations:** 1 Serviço de Neuro-oftalmologia, Instituto de Olhos, Faculdade Ciências Médicas de Minas Gerais, Belo Horizonte, MG, Brazil; 2 Department of Ophthalmology, Otorhinolaryngology, and Head and Neck Surgery, Faculdade de Medicina de Ribeirão Preto, Universidade de São Paulo, Ribeirão Preto, SP, Brazil

Dear Editor,

In ophthalmological practice, most diseases allow elective treatment; however, some conditions require prompt management, such as acute retinal detachment, angle closure glaucoma, corneal ulcer, and retinal necrosis. In parallel, giant cell arteritis (GCA) is one of the few life-threatening diseases that eye doctors must deal with and should therefore be treated as a medical emergency.

GCA is a common form of systemic vasculitis that typically affects patients aged >50 y, and unfortunately, GCA is a common cause of blindness among the elderly, probably due to late diagnosis or lack of proper investigation^(1)^.

In patients with GCA, ophthalmologist may detect new onset of diplopia, episodes of amaurosis fugax, severe loss of vision, and blindness in association to the clinical findings such as ocular misalignment, central retina artery occlusion, and pale optic disc edema, with or without retinal exudates. Furthermore, patients under suspicion should be evaluated in more detail, beyond eye signs and symptoms, and physicians should investigate for malaise, fatigue, fever, headaches, temporal and scalp tenderness, loss of weight, jaw claudication, new onset of sadness and/or depression. Additionally, laboratory tests, such as complete blood count, C-reactive protein (CRP) and erythrocyte sedimentation rate (ESR) should always be performed. It is not expected that the disease starts acutely, and the patient’s perception of the systemic symptoms usually starts weeks before eye involvement^(2)^.

In parallel, COVID-19 patients can present with acute onset headache, fever, fatigue, myalgia, scalp and temporal artery tenderness, difficulty in chewing, transient visual loss, weight loss, dysphagia, or elevation of inflammatory markers, such as serum concentration of CRP and ESR^(1)^. Thus, the “GCA-like” features of SARS-CoV-2 infection certainly confound clinicians managing GCA and/or COVID-19 patients, and recently, Riera-Marti et al. even suggest COVID-19 infection as a trigger for GCA^(3)^.

As a consequence, the COVID-19 pandemic has presented as a challenge in the evaluation of patients with suspected GCA owing to the possible overlapping signs and symptoms of these two clinical conditions^(4)^. It is noteworthy that a brief PubMed search revealed observations from several parts of the World that indicate an association between COVID-19 and increasing risk of GCA^(1-5)^.

Seemingly, we have a clear impression of increasing number of GCA cases in Belo Horizonte during the pandemic. Comparing our numbers to a previous report, in the Fondation Adolphe de Rothschild Hospital, in Paris-France, they observed 17 cases during the COVID-19 pandemic; however, only 10 cases were reported during the same period in 2019, corresponding to an increase of 70% in the incidence^(5)^. We diagnosed 7 cases of GCA during the pandemic time, but only 2 in 2019 (250% increase). Table 1 shows the demographic and clinical data from our 7 patients.

**Table 1:** Clinical features of GCA patients during COVID-19 pandemic (from July 20 to April 21; n=7)

#	Gender; age (years)	Initial symptoms	Time to diagnosis	Final visual outcome	↑ ESR and/or ↑ CRP	TAB	PCR-RT SARS-CoV-2 #
1	F; 76	Fever; headache	2 weeks	OD: 20/100; OS: HM	**+**	**+**	Negative
2	F; 81	Diplopia	2 weeks	OD: HM; OS: 20/20	**+**	**+**	Negative
3	F; 82	Headache	1 month	OD: 20/25; OS: 20/400^*^	**+**	Unknown	Unknown
4	F; 87	Diplopia	1 week	OD: NLP; OS: NLP	**+**	**+**	Positive
5	M; 84	Jaw claudication	2 weeks	OD: HM; OS:20/20	**+**	**+**	Negative
6	F; 86	Headache	1 month	OD: HM; OS: NLP	**+**	- ^***^	Negative
7	F; 74	Diplopia	1 week	OD: 20/50; OS: 20/20^**^	-	**+**	Negative

* = remaining visual field <5 degrees OU (Goldmann visual field);

** = visual acuity not affected OU (diplopia only).

*** = done after 6 weeks of steroids; # Test done only once. ESR= erythrocyte sedimentation rate; CRP= C-reactive protein; HM= hand motion; NLP= no light perception; TAB= temporal artery biopsy.

Most of our new GCA cases had no clear association to SARS-CoV-2 infection, and in only one case, the real time *polymerase chain reaction* (RT-PCR) was positive (Table 1). In the report of the higher incidence rate of GCA during the COVID-19 pandemic, the status of SARS-CoV-2 infection was not provided^(5)^.

The mechanism of association between COVID-19 infection and a systemic vasculitis opens several questions; however, there is a hypothesis that it might mimic GCA or that SARS-CoV-2 infection might directly induce GCA via COVID-19-associated endothelial dysfunction^(2)^.

Other neuro-ophthalmologic disorders, such as, optic neuritis, cranial nerve palsies, Adie’s pupil, parainfectious encephalitis, stroke, idiopathic intracranial hypertension, ocular myasthenia, and Miller fisher syndrome are already associated with COVID-19; however, fewer studies have reported on the association of COVID-19 and GCA; therefore, we would like to alert ophthalmologists to suspect GCA in all patients aged >50 y that present with typical symptoms in one or both eyes, reminding that a prompt diagnosis is the key to avoid irreversible loss of vision.

In short, the diagnosis of GCA has never been simple, owing to the variability of presentation of its diverse, and sometimes misleading visual, ocular, systemic and laboratory findings. In recent times, with the overlap of COVID-19 symptom, ophthalmologists should pay more attention to raise clinical suspicion and provide accurate investigation and prompt treatment for their patients.
